# The effectiveness of Facebook as a social network intervention to increase physical activity in Chinese young adults

**DOI:** 10.3389/fpubh.2022.912327

**Published:** 2022-07-22

**Authors:** Patrick W. C. Lau, J. J. Wang, Lynda L. Ransdell, Lei Shi

**Affiliations:** ^1^Department of Sport, Physical Education and Health, Hong Kong Baptist University, Hong Kong, China; ^2^Laboratory of Exercise Science and Health, Beijing Normal University-Hong Kong Baptist University United International College (UIC), Zhuhai, China; ^3^National Fitness and Scientific Exercise Research Center, China Institute of Sport Science, Beijing, China; ^4^Department of Kinesiology, Boise State University, Boise, ID, United States

**Keywords:** social network, social media, Facebook, e-health, physical activity, intervention

## Abstract

**Introduction:**

Facebook, one of the worlds' most popular online social networks, is easy to access and cost-effective. Its use to enhance individual's physical activity (PA) participation should be examined. This research reviews the effectiveness and successful features of Facebook for influencing PA behaviors in young adults (Study 1), and empirically examines the efficacy of the features purported in increase PA *via* a Facebook intervention (Study 2).

**Methods:**

In Study 1, PubMed, Medline, SPORT Discus, ERIC, and Embase were searched for articles that identified successful features and effectiveness of Facebook PA interventions published between January 2005 and February 2022. In Study 2, a 4-week Facebook PA intervention with University students was conducted using features identified in Study 1. The PA behaviors with objective (ActiGraph) and subjective (questionnaire) measures, perceived PA level, stage of readiness, effectiveness, and efficiency of Facebook were examined.

**Results:**

Study 1 concluded that the most effective strategies for producing significant PA changes in young adults using a PA Facebook intervention included the following: Adding behavior modification (goal setting and self-monitoring), using influence agents, recruiting members of an existing network with the snowball technique, being attentive to group size, enhancing social support with motivational quotes, interactive posts, opinion polls, increasing tailored feedback, and providing educational information. Study 2 found no significant difference in PA between the intervention and the control groups, as measured objectively, but the subjective reporting of PA behavior was higher in the intervention group. Compared to the control group, the Facebook PA intervention group reported more positive change in perceived stage of readiness in PA participation, commuting type, sport type, sport venue, sport emotion, and fast breathing or sweating. When features were ranked by the Facebook PA intervention group, motivation (supports from your friends) and tailored feedback (the responses from your friends are really personal and fits you) were the top two ranked features.

**Conclusion:**

The use of influence agents in the Facebook PA intervention could address exercise preference and facilitate higher program engagement. Significant differences related to commuting type, sport types, sport venue barriers, and exercise intensity across groups were noteworthy and warrant additional investigation in the future.

## Introduction

Insufficient physical activity (PA) represents a major contributing factor to morbidity and mortality across the world (1). Physical inactivity is positively correlated with many chronic diseases, including diabetes, heart disease, physical and mental health problems, and even certain types of cancer (2). It is well established that when youth participate in PA, it leads to many health-related benefits, including a higher likelihood of maintaining PA behaviors through adulthood (3). Unfortunately, studies have shown that during the transition period from adolescence to adulthood, PA levels decrease sharply (4). When using 60 min of moderate-to-vigorous physical activity (MVPA) per day as a criterion, the prior studies have concluded that <20% of American adolescents (5), <19% of Australian children aged 5–17 years (6), and <8.4% of Hong Kong young adults meet this recommendation (7). It is clear that the physical inactivity in young adults is a worldwide phenomenon, with life-long health implications.

Traditionally, interventions designed to increase PA participation in young adults are implemented through face-to-face delivery. Most of these interventions are effective, however, there are limitations in terms of reach, maintenance accessibility, and cost (8). The US Centers for Disease Control and Prevention found that lack of social support is a major barrier to an individual's PA participation (9). Social media may be one potential way to improve social support for PA, as American teens spend an average of 9 h per day on social media (not including time for homework) (10). The American Academy of Child and Adolescent Psychiatry (10) states that some potential benefits of social media include the following: Staying connected to friends, meeting new friends with shared interests, finding community and support for specific activities, and exploring or expressing themselves. Channeling the positive benefits of social media is more important than ever due to the current COVID-19 pandemic, which has changed the way we all interact.

Researchers who have examined increasing PA through social media have concluded that Facebook is effective and efficient in terms of recruiting participants, follow-up investigation, social support, health information delivery, and intervention delivery (11–13). These studies have been conducted with different populations including patients, disabled children, pregnant woman and obese children, and Facebook works either alone or with other e-technologies such as email, websites, or text messaging (14–17).

The rapid growth of social media use in our youth, along with the current COVID-19 pandemic (which has changed the way humans interact), provide a natural forum for online social networks to deliver health behavior change interventions. Recently, the researchers have focused on developing online social networks, as their use has been significantly growing from 90 min/day in 2012 to 147 min/day in 2022 (18). Some of the attractive features of online social networks include large audiences, existing contacts, high levels of engagement and retention, and cost-effectiveness (6, 19). Online social networks, which encourage sharing and openness, have significant “push” effects compared with traditional information dissemination and communication technology (e.g., websites and SMS). Facebook, well-known as the largest social networking platform in the world, has 1.8 billion users each month (20). In Hong Kong (HK), nearly 64% of the total population is active on social media, and Facebook is the top-ranked platform for social networking there. More than 3.1 million people log on to Facebook every day, over 50% of the HK population uses Facebook, and users spend an average of 30 min during each visit (21).

Recently, the researchers have begun to examine using Facebook to connect social media users to online social networks. Several studies have compiled information, made programming recommendations, and presented suggestions for future research. A systematic review by Maher et al. (8) examined the impact of online social networks on general health behaviors and concluded that 9 of the 10 studies reported improvements in health behavior change; however, changes were small and non-significant, participant attrition was high, and fidelity was low (typically between 5–15%). In terms of connecting social networks to PA resources, Nakhasi et al. (9) concluded that 9 of 13 social health activity networks (69%) connected social media users to PA partners.

When PA behaviors were specifically examined, Joseph et al. (22) reviewed 10 studies and found that 70% of the studies (*n* = 7 out of 10) reported significant within- or between-group differences for at least 1 PA outcome. A more recent review by Goodyear et al. (11) revealed that positive changes in PA behavior resulted from the accessibility of health information and social interaction on the Facebook intervention. Günther et al. (12) reported that more than one-third of the intervention studies showed positive effects on PA.

The researchers have also begun to examine which aspects of e-health interventions facilitate behavior change. Rose et al. (23) reported that significant PA behavior change was seen in an e-Health intervention that used Facebook when it consisted of goal setting, self-monitoring, and health education. In summary, evidence suggests that social media may be a promising tool for increasing PA.

Although some research has been done, there are many unanswered questions. To gain a more comprehensive view of Facebook's potential contribution to changing PA behavior in a specific, impressionable population (e.g., Chinese young adults), this research consisted of the following two studies: Study 1 reviewed the literature and identified the features of successful Facebook interventions to increase PA. Study 2 was an empirical study that tested the efficacy of the features purported to increase PA *via* a Facebook intervention. This study enhances knowledge-related to changing PA behavior using a social media (Facebook) intervention for young adults.

## Study 1

### Methods

PubMed, Medline, SPORTDiscus, ERIC, and Embase were searched from January 2005 to February 2022 using keywords “Facebook/OR social network/OR social media/OR Twitter/OR web /OR internet” combined with “physical activity/OR physical fitness.” The eligibility criteria were as follows: (1) Experimental studies and systematic reviews reported in peer-reviewed journals or as peer-reviewed full conference papers, (2) studies using an online social network either wholly or in part to increase PA, (3) participants were adults and children regardless of their health status, and (4) the intervention had to target PA level and measure related outcomes.

### Results

**Figure 1:**
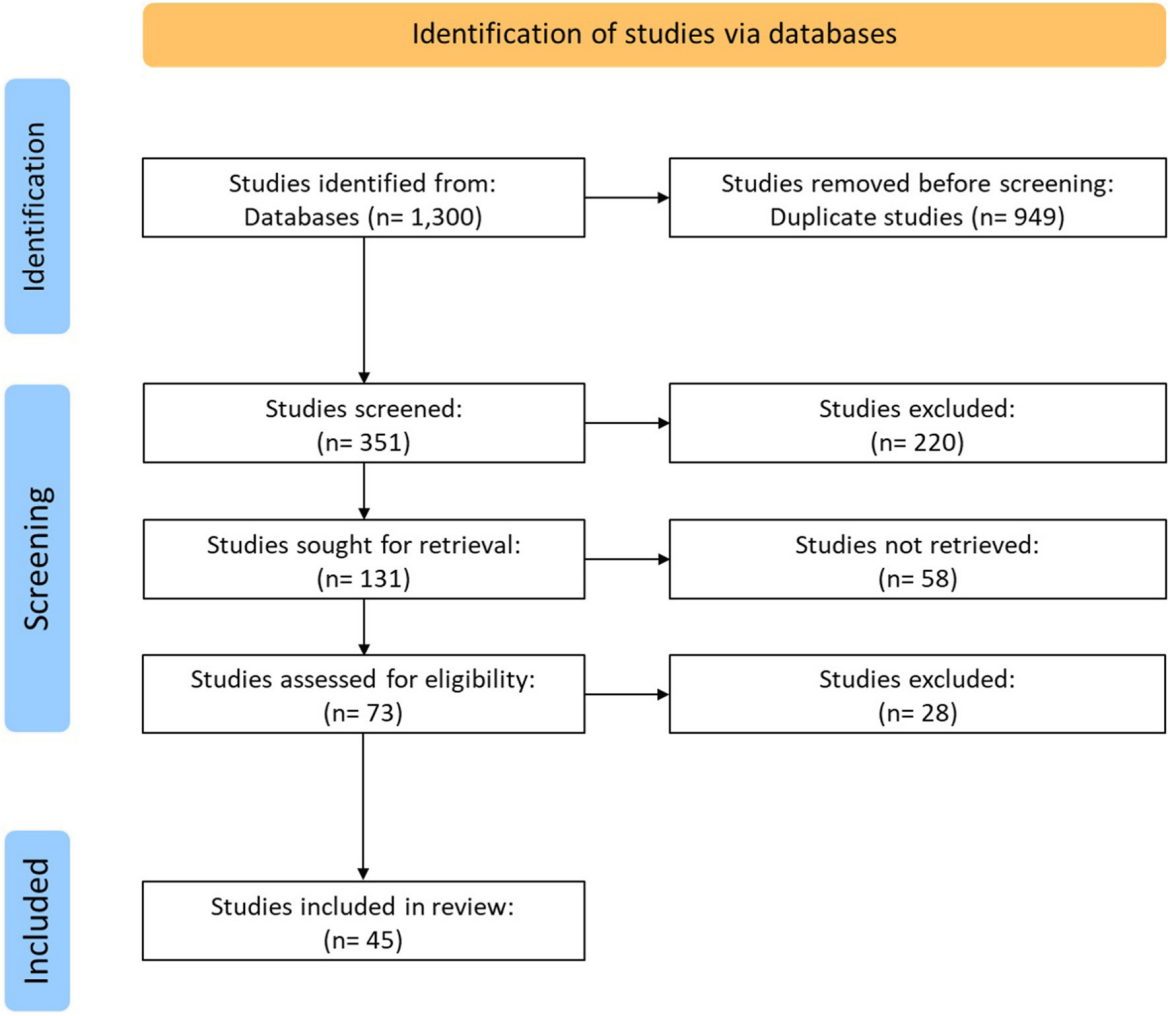
Selection diagram of literature research.

Of the 1,300 initial search results, 45 met the eligibility criteria, and 38 of those were original research studies or study protocols, and 7 were literature reviews (Figure 1).

#### The effectiveness of social network interventions on PA behavior

Of the 38 original studies, 31 (81.6%) showed an increase in self-reported (subjective) PA level; conversely, few of these studies showed an increase in objective PA as measured by a device such as an accelerometer. Specifically, Fjeldsoe et al. (24) reported that MobileMums, an intervention for increasing PA in women, increased participants' self-reported MVPA duration and frequency, whereas no effects were observed in accelerometer-derived MVPA. Edney et al. (25) also found that the social network PA intervention increased self-reported MVPA but not objective PA among adults. Clearly, more research is needed in this area to discern why this discrepancy occurs between objective and subjective measures of PA.

A second finding was that positive results mainly occurred in light intensity PA or walking time compared to vigorous PA. For example, in a Facebook-based PA intervention for insufficient active adults, Maher et al. (8) found that after 8-week follow-up, the intervention group increased walking time by 155 min/week compared to controls, and there were no significant changes in vigorous PA. Joseph et al. (26), who designed an 8-week e-intervention to increase PA in 29 African American women, also observed that participants in the experiment group decreased sedentary time and increased light and moderate intensity instead of vigorous PA.

Third, in a couple studies, participants' awareness, motivation, perceived behavior control, and satisfaction increased after a 3–4-months intervention (25, 27). Specifically, program adherence/engagement increased significantly after using social network intervention (13, 20, 28).

Taken together, these findings indicate that social media may have an impact on the adoption and maintenance of light intensity PA, and on the psychological aspects of PA adherence and engagement.

#### The effectiveness of Facebook PA interventions among young adults

The results were mixed relative to the impact of Facebook PA interventions on young adult's PA behaviors although most of the findings were similar to what was found in Section The effectiveness of social network interventions on PA behavior. Most studies showed increases in self-reported PA as a result of participating in a Facebook PA intervention. For example, in a pilot RCT designed to increase low-active young adult's PA, significant improvements were found over time on self-reported weekly leisure–time PA, although no interaction was observed between groups or on objectively measured outcomes (19). Pope et al. (13) also reported that college students increased their MVPA after a 12-week Facebook intervention.

Rote et al. (29) evaluated the efficacy of a walking intervention *vs*. control group using Facebook among female college freshmen. Compared to the control group, the walking intervention participants reported a significant increase in objectively measured (pedometer) walking (e.g., approximately 1.5 miles/day) and a decrease in waist circumference. Social support was highlighted as a pivotal element in this successful intervention (29).

Still other studies reported that, although there was no significant improvement in PA level, sedentary time decreased. For example, in a 12-week, Facebook-based lifestyle intervention designed for overweight and obese adolescents, the intervention did not increase participants' PA level, but sedentary time decreased 11% on the weekdays after the intervention. This might indicate that when overweight and obese adolescents begin to get active, they may first reduce daily sedentary time rather than participate in high-intensity PA, due to the fact that their attitudes to be active may be more negative compared to their normal weight counterparts (3).

Kernot et al. (20) conducted a 6-months Facebook PA intervention in postpartum women, and found no differences in objective MVPA or self-reported walking after completing the intervention. These inconsistent findings point to the need to collect more data to examine the effectiveness of using social networks and Facebook for increasing/enhancing PA.

#### Effective features in Facebook

After reviewing prior research related to using social network interventions to increase/enhance PA, it is important to summarize features that should help change a young adult's PA behavior through a Facebook intervention.

1) **Behavior modification strategies** that have been previously successful in Facebook PA interventions include cognitive strategies such as goal setting and self-monitoring (23, 30, 31).
2) **Influence agents** or a small group of individuals who promote behavior change and provide support when/where needed, may be effective for facilitating behavior change. Van Woudenberg et al. (32) concluded that a small group of individuals (influence agents) is effective for promoting health-related behaviors in a social network intervention.
3) The **snowball method** should be used to recruit “influence agents” into an intervention group as it may improve effectiveness (8, 33–35). Peers may be more influenced by peers than by strangers. Cavallo et al. (36) concluded that a failure to facilitate effective social support from their Facebook group may have been due to the unfamiliar members (36). Other factors to consider are that there may be an ideal number of influence agents and an ideal group size. Rote et al. (29) specified that the ideal group size is 5–9, because if the group is larger, information may be excessive, and if the group size is smaller, social support may be insufficient. Finally, it is likely that recruiting influence agents using the snowball method may attract individuals with similar characteristics, which may enhance program success.
4) **Social support** defined as enhancing companionship, esteem, and encouragement (33), is at the core of a social network PA intervention (29). Irvin et al. (37) stated that a behavior change intervention focused on enhancing group dynamics is effective for facilitating social support and consequently improving PA behavior. Daily interactive posts, motivational quotes, and opinion polls are important for enhancing social support among Facebook members (38). Goodyear et al. (11) suggested that group chats and frequent interaction were the main characteristics of social media interventions to produce positive PA behavior change.
5) **Tailored feedback** consists of providing participants with individualized information based on their preferences and motivational incentives. Nishiwaki, et al. (39) designed an intervention study where an observer communicated with each participant and provided tailored feedback; this effort resulted in a significant increase in moderate PA.
6) **Educational information**, which consists of various behavior management skills including PA information links (definitions, benefits, and guidelines), goal setting strategies, setting individual expectations, overcoming barriers and maintaining behaviors, is another essential strategy for a successful social network PA intervention (11, 23, 38, 40).

## Study 2

### Methods

#### Study design and participants

A 4-week Facebook PA intervention was designed using the features recommended in Study 1. The intervention was implemented with college students. Intervention effectiveness was evaluated using several measures, collected pre- and post-intervention, including objective PA (accelerometer), subjective PA (sport type, venue, duration and intensity, and commuting type), and psychological aspects of PA participation (stage of motivational readiness; sport emotion). The snowball sampling was used to recruit participants. In the initial stage, physical education (PE) major students were selected as the influence agents, also known as the captains. The captains then invited their friends to join his/her group. In total, 62 young adults, aged 18–25 years agreed to participate in this study after signing the consent form. There were 5 intervention groups between 5 and 9 participants, and 3 control groups between 10 and 11 participants.

The PA level of the participants from intervention and control group was measured at baseline (pre-intervention) and upon completion of the intervention (post-intervention). During the intervention, each group used one non-public Facebook Page which could only be seen by captain and the group members, as well as related research staff. The intervention group members received education links and intervention messages on the Facebook page posted by the captains, and were required to complete a 14-item questionnaire on the page each day. Captions could have a personalized page name and photos could be added to the page to make it more attractive. In addition, intervention group captains participated in discussions among his/her group members about intervention contents, and they were encouraged to share their experiences and feelings. Intervention messages were sent on a daily basis, and were designed to promote PA levels through goal setting, peer support, motivational quotes, and tailored feedback. The participants from the control group received no such intervention messages and had no group captain or discussion; they only finished the questionnaires.

#### Measurements

Two types of measurements were used in the study as follows: Objective measurements by ActiGraph and subjective measurements by self-reported questionnaire, which were collected on the Facebook page.

#### Objective PA measure

The PA level at baseline and post-test was measured using an activity monitor from ActiGraph (Pensacola, FL). The models GT3X, GT3X+, and wGT3X-BT were used, and initialized at an epoch of 5 s (GT3X) and a sampling rate of 30 Hz (GT3X+, wGT3X-BT). Each participant carried the same accelerometer over a 2–3-week period. At least 3 valid weekdays and 1 valid weekend day were required for an eligible wearing. For each valid day, a wearing time of more than or 480 min was required. A non-wear period was defined as 60 min minimum length with a spike tolerance of 2 min. Vector magnitude was also applied. For setting of cut points and MVPA, there were four categories: Sedentary (0–99 cpm); light (100–2,019 cpm); moderate (2,020–5,998 cpm); and vigorous (5,999 cpm and above). The participants were asked to wear the accelerometers for an additional time if their first wearing data were unqualified.

To collect additional objective PA information, and verify step count stated in the questionnaire, a free version mobile App named Accupedo pedometer was used after getting approval from the company. The App was selected due to the fact that it was one of the most downloaded pedometer applications, and has shown good accuracy and compatibility with different mobile phones (41).

#### Self-reported questionnaire

The self-constructed and self-report questionnaire aimed to collect daily physical activity-related information such as commuting type, sport type, sport venue, sport duration, and intensity. The participants were asked to complete this questionnaire daily *via* their Facebook account. A pre-post questionnaire covering psychological aspects of PA (e.g., perceived PA level, stage of readiness in PA participation, effectiveness and efficiency, and attractive features) was also conducted to the both groups.

#### Statistical analyses

The ActiGraph data were analyzed by ANCOVA with group (intervention and control) as the factor and baseline PA as the covariate. Between-group mean difference and its 95% confidence interval (CI) were calculated after adjusting the baseline difference between intervention and control groups. The differences of subjective outcomes between intervention and control groups were tested week by week. Independent *t*-tests were used for continuous variables, and Chi-squared tests were used for categorical variables; *p* < 0.05 was used as the significance level.

### Results

A sample of 56 young adults aged 18–25 years joined this study and 49 (38.8% male) of the participants completed the post-test. Of this group, 24 participants were in the intervention group and 25 were in the control group. The average age was 21.5 ± 1.68 years. No gender or age differences were found between the intervention and control groups.

No significant difference was found in the objective/ActiGraph measurements between intervention and control groups (Table 1), but participants in the intervention group self-reported a 33% increase (before intervention: 32%, after intervention: 65%) in meeting the ACSM PA guideline, compared to a 10% perceived increase (before 45%, after 55%) in the control group. Also, 23 and 10% PA level improvement were reported by both intervention and control groups.

**Table 1:** Actigraph measurements between intervention and control groups.

**Outcomes**	**Intervention Mean** ±**SD**	**Control Mean** ±**SD**	**Between-group Mean difference (95% CI)**	***P***
Average MVPA per day				
Baseline (28 vs. 28)	62.52 ± 24.41	72.01 ± 25.99		
Post (24 vs. 25)	57.42 ± 19.57	58.42 ± 24.59	2.41 (−8.63, 13.45)	0.662
Percent of time in MVPA, %				
Baseline (28 vs. 28)	8.66 ± 3.47	9.83 ± 3.13		
Post (24 vs. 25)	8.24 ± 3.04	8.04 ± 2.99	0.74 (−0.83, 2.15)	0.377
Average vector magnitude of all 3 Axis				
Baseline (28 vs. 28)	64.51 ± 18.76	80.89 ± 24.41		
Post (24 vs. 25)	67.99 ± 17.19	63.18 ± 20.00	9.21 (−1.47, 19.90)	0.089
Average step counts per day				
Baseline (28 vs. 28)	9,560 ± 2,600	8,984 ± 2,202		
Post (24 vs. 25)	9,366 ± 2,490	8,104 ± 3,301	1,019 (−630, 2,668)	0.220

Table 2 describes the change in sport duration and step counts between the two groups. No difference was found in sport duration between two groups, neither on the weekdays nor during the weekend. The intervention group reported significantly more steps than the control group in the first week, either in the step counts for the weekend (between-group mean difference: 4,357, 95% CI: 2,167–6,548, *p* < 0.001) or for the total step counts from the whole week (between-group mean difference: 1,892, 95% CI: 217–3,568, *p* = 0.028).

**Table 2:** Differences of sport duration and step count between two groups.

**Outcomes**	**Intervention Mean** ±**SD**	**Control Mean** ±**SD**	**Mean difference (95% CI)**	***P***
**Sport duration (total)**				
Week 1	74.11 ± 87.16	81.93 ± 124.47	−7.82 (−65.39, 49.75)	0.786
Week 2	57.07 ± 69.65	54.30 ± 71.32	2.78 (−35.35, 40.90)	0.884
Week 3	30.39 ± 33.98	36.07 ± 71.18	−5.68 (−35.84, 24.48)	0.705
Week 4	32.88 ± 38.54	17.25 ± 26.74	15.63 (−3.16, 34.42)	0.101
**Sport duration (weekday)**				
Week 1	61.14 ± 90.46	77.39 ± 129.39	−16.25 (−76.07, 43.57)	0.588
Week 2	56.25 ± 72.44	58.96 ± 93.34	−2.71 (−47.80, 42.38)	0.904
Week 3	25.39 ± 33.35	33.61 ± 65.16	−8.21 (−36.17, 19.74)	0.556
Week 4	30.79 ± 36.10	13.71 ± 25.32	17.08 (−0.58, 34.73)	0.058
**Sport duration (weekend)**				
Week 1	105.39 ± 101.79	90.89 ± 147.12	14.50 (−53.28, 82.28)	0.670
Week 2	59.43 ± 76.46	46.46 ± 82.03	12.97 (−30.31, 56.24)	0.550
Week 3	41.54 ± 53.41	38.82 ± 106.42	2.72 (−42.57, 48.01)	0.905
Week 4	37.82 ± 58.89	24.58 ± 46.95	13.24 (−16.78, 43.26)	0.380
**Step count (total)**				
Week 1	9,301 ± 3,459	7,409 ± 2,756	1,892 (217, 3,568)	0.028
Week 2	9,182 ± 3,440	7,921 ± 2,762	1,261 (−410, 2,933)	0.136
Week 3	8,724 ± 3,175	8,082 ± 2,927	642 (−994, 2,278)	0.435
Week 4	8,724 ± 2,777	7,916 ± 3,367	808 (−904, 2,519)	0.348
**Step count (weekday)**				
Week 1	8,651 ± 3,612	7,747 ± 2,835	905 (−835, 2,644)	0.302
Week 2	9,119 ± 3,699	7,958 ± 2,941	1,161 (−629, 2,951)	0.199
Week 3	8,679 ± 3,713	8,503 ± 2,934	176 (−1,617, 1,969)	0.844
Week 4	9,028 ± 3,109	7,856 ± 3,091	1,172 (−561, 2,904)	0.180
**Step count (weekend)**				
Week 1	10,930 ± 4,713	6,572 ± 3,348	4,357 (2,167, 6,548)	<0.001
Week 2	9,235 ± 4,207	7,549 ± 4,206	1,687 (−589, 3,962)	0.143
Week 3	8,836 ± 3,193	6,916 ± 3,927	1,920 (−13, 3,852)	0.051
Week 4	8,144 ± 3,965	8,062 ± 5,087	82 (−2,441, 2,605)	0.948


**Figure 2:**
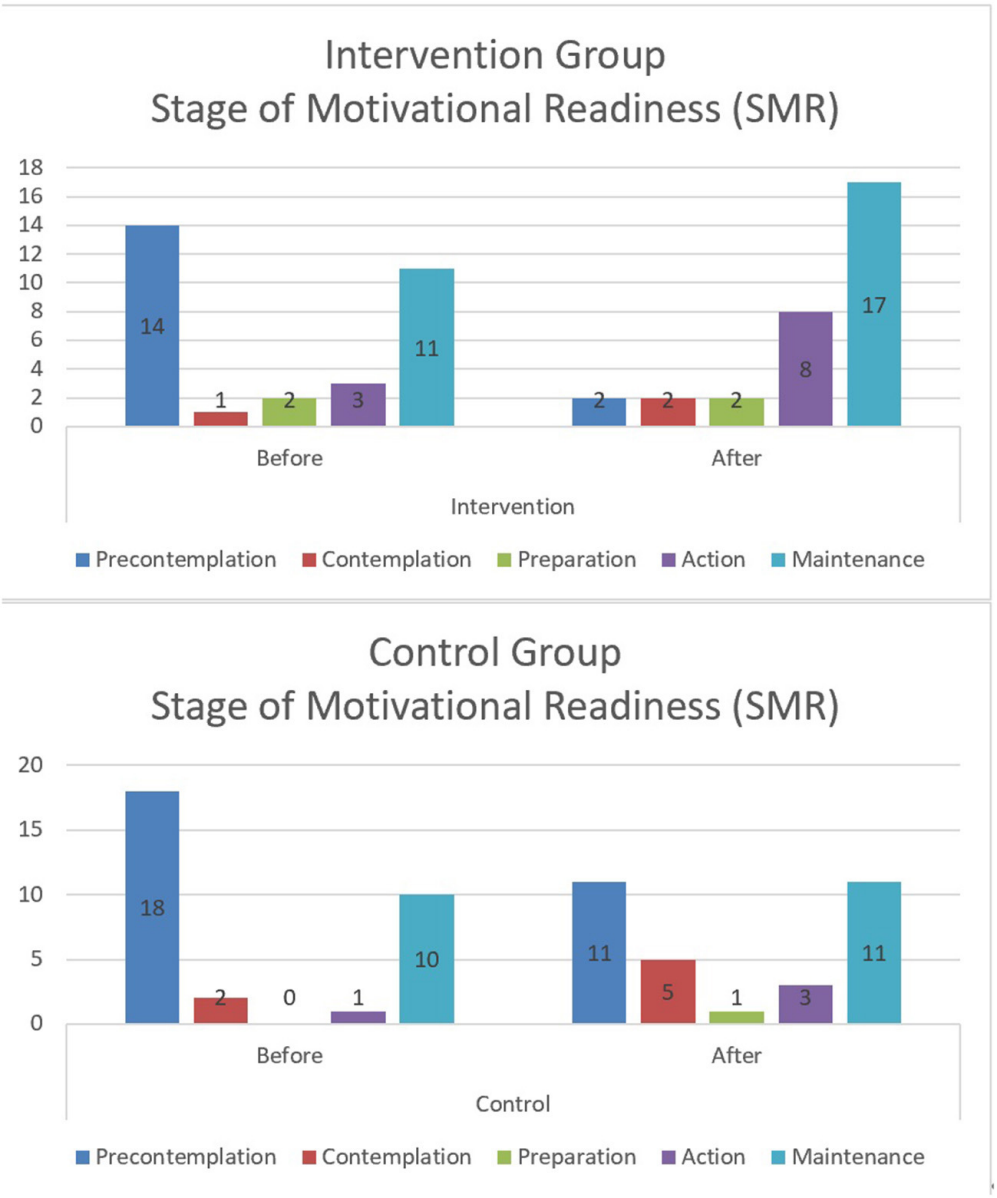
Changes in stage of motivational readiness between two groups.

Finally, more positive changes in perceived stage of readiness in PA participation were reported from the pre-contemplation to action/maintenance stages in the intervention compared to control group (Figure 2). These results indicated a clear discrepancy between objective and subjective PA measurement.

Tables 3–7 describe between and within group changes in commuting type, sport type, sport venue, sport emotion, and whether the participant reported fast breathing or sweating.

**Table 3:** Differences of commuting type between two groups.

**Outcomes**		**Total *n* (%)**	**Intervention *n* (%)**	**Control *n* (%)**	**χ^2^ value**	***P***
**Commuting type**						
Week 1	None	44 (11.5%)	19 (9.8%)	25 (13.2%)	8.363	0.039
	Walk	133 (34.7%)	60 (30.9%)	73 (38.6%)		
	Bike	3 (0.8%)	3 (1.5%)	0 (0.0%)		
	Public transport	203 (53.0%)	112 (57.7%)	91 (48.1%)		
Week 2	None	52 (13.4%)	19 (9.7%)	33 (17.2%)	6.775	0.034
	Walk	113 (29.2%)	53 (27.2%)	60 (31.3%)		
	Bike	0 (0.0%)	0 (0.0%)	0 (0.0%)		
	Public transport	222 (57.4%)	123 (63.1%)	99 (51.6%)		
Week 3	None	50 (13.3%)	22 (11.4%)	28 (15.2%)	5.135	0.162
	Walk	119 (31.6%)	55 (28.5%)	64 (34.8%)		
	Bike	1 (0.3%)	1 (0.5%)	0 (0.0%)		
	Public transport	207 (54.9%)	115 (59.6%)	92 (50.0%)		
Week 4	None	41 (10.8%)	19 (9.1%)	22 (12.7%)	3.469	0.177
	Walk	127 (33.3%)	64 (30.8%)	63 (36.4%)		
	Bike	0 (0.0%)	0 (0.0%)	0 (0.0%)		
	Public transport	213 (55.9%)	125 (60.1%)	88 (50.9%)		

**Table 4:** Differences of sport type between two groups.

**Outcomes**		**Total** ***n*** **(%)**	**Intervention** ***n*** **(%)**	**Control** ***n*** **(%)**	χ^2^ **value**	***P***
**Sport type**						
Week 1	None	167 (42.9%)	65 (33.3%)	102 (52.6%)	22.082	<0.001
	Walking	139 (35.7%)	73 (37.4%)	66 (34.0%)		
	Jogging	14 (3.6%)	12 (6.2%)	2 (1.0%)		
	Others^a^	69 (17.7%)	45 (23.1%)	24 (12.4%)		
Week 2	None	203 (53.0%)	76 (39.6%)	127 (66.5%)	30.222	<0.001
	Walking	102 (26.6%)	62 (32.3%)	40 (20.9%)		
	Jogging	12 (3.1%)	10 (5.2%)	2 (1.0%)		
	Others^a^	66 (17.2%)	44 (22.9%)	22 (11.5%)		
Week 3	None	202 (53.9%)	70 (37.2%)	132 (70.6%)	48.755	<0.001
	Walking	92 (24.5%)	56 (29.8%)	36 (19.3%)		
	Jogging	9 (2.4%)	8 (4.3%)	1 (0.5%)		
	Others^a^	72 (19.2%)	54 (28.7%)	18 (9.6%)		
Week 4	None	227 (59.1%)	102 (48.1%)	125 (72.7%)	25.970	<0.001
	Walking	76 (19.8%)	50 (23.6%)	26 (15.1%)		
	Jogging	15 (3.9%)	13 (6.1%)	2 (1.2%)		
	Others^a^	66 (17.2%)	47 (22.2%)	19 (11.0%)		

a *“Others” included ball game, water activity, muscle training and others*.

**Table 5:** Differences of sport venue between two groups.

**Outcomes**		**Total** ***n*** **(%)**	**Intervention** ***n*** **(%)**	**Control** ***n*** **(%)**	χ^2^ **value**	***P***
**Sport venue**						
Week 1	None	194 (52.4%)	76 (39.6%)	118 (66.3%)	48.388	<0.001
	Home	4 (1.1%)	1 (0.5%)	3 (1.7%)		
	School	25 (6.8%)	7 (3.6%)	18 (10.1%)		
	Others^a^	147 (39.7%)	108 (56.3%)	39 (21.9%)		
Week 2	None	216 (58.1%)	82 (43.2%)	134 (73.6%)	51.865	<0.001
	Home	7 (1.9%)	6 (3.2%)	1 (0.5%)		
	School	14 (3.8%)	3 (1.6%)	11 (6.0%)		
	Others^a^	135 (36.3%)	99 (52.1%)	36 (19.8%)		
Week 3	None	244 (64.4%)	95 (48.7%)	149 (81.0%)	60.847	<0.001
	Home	23 (6.1%)	22 (11.3%)	1 (0.5%)		
	School	6 (1.6%)	1 (0.5%)	5 (2.7%)		
	Others^a^	106 (28.0%)	77 (39.5%)	29 (15.8%)		
Week 4	None	247 (64.8%)	106 (50.5%)	141 (82.5%)	55.971	<0.001
	Home	18 (4.7%)	16 (7.6%)	2 (1.2%)		
	School	6 (1.6%)	1 (0.5%)	5 (2.9%)		
	Others^a^	110 (28.9%)	87 (41.4%)	23 (13.5%)		

a *“Others” included sport hall, playground, gym, swimming pool, park and others*.

**Table 6:** Differences of emotion between two groups.

**Outcomes**		**Total *n* (%)**	**Intervention *n* (%)**	**Control *n* (%)**	**χ^2^ value**	***P***
**Emotion**						
Week 1	None	135 (35.0%)	53 (27.3%)	82 (42.7%)	12.019	0.007
	Positive^a^	214 (55.4%)	124 (63.9%)	90 (46.9%)		
	Negative^b^	32 (8.3%)	15 (7.7%)	17 (8.9%)		
	Others	5 (1.3%)	2 (1.0%)	3 (1.6%)		
Week 2	None	152 (39.4%)	52 (27.2%)	100 (51.3%)	25.889	<0.001
	Positive^a^	190 (49.2%)	117 (61.3%)	73 (37.4%)		
	Negative^b^	39 (10.1%)	19 (9.9%)	20 (10.3%)		
	Others	5 (1.3%)	3 (1.6%)	2 (1.0%)		
Week 3	None	187 (50.4%)	86 (45.7%)	101 (55.2%)	6.614	0.085
	Positive^a^	148 (39.9%)	85 (45.2%)	63 (34.4%)		
	Negative^b^	29 (7.8%)	12 (6.4%)	17 (9.3%)		
	Others	7 (1.9%)	5 (2.7%)	2 (1.1%)		
Week 4	None	184 (48.5%)	89 (42.6%)	95 (55.9%)	16.524	0.001
	Positive^a^	153 (40.4%)	103 (49.3%)	50 (29.4%)		
	Negative^b^	39 (10.3%)	16 (7.7%)	23 (13.5%)		
	Others	3 (0.8%)	1 (0.5%)	2 (1.2%)		

a *“Positive” emotion in sport included “happy” and “relax”. ^b^ “Negative” emotion in sport included “sad”, “worried”, and “angry”*.

**Table 7:** Differences of fast breathing and sweating between two groups.

**Outcomes**		**Total** ***n*** **(%)**	**Intervention** ***n*** **(%)**	**Control** ***n*** **(%)**	χ^2^ **value**	***P***
**Breathing fast**						
Week 1	No	299 (76.3%)	129 (65.8%)	170 (86.7%)	23.697	<0.001
	Yes	93 (23.7%)	67 (34.2%)	26 (13.3%)		
Week 2	No	293 (76.3%)	127 (65.1%)	166 (87.8%)	27.357	<0.001
	Yes	91 (23.7%)	68 (34.9%)	23 (12.2%)		
Week 3	No	292 (76.8%)	134 (68.0%)	158 (86.3%)	17.890	<0.001
	Yes	88 (23.2%)	63 (32.0%)	25 (13.7%)		
Week 4	No	292 (75.8%)	141 (66.5%)	151 (87.3%)	22.439	<0.001
	Yes	93 (24.2%)	71 (33.5%)	22 (12.7%)		
Sweating						
Week 1	No	259 (66.9%)	96 (49.7%)	163 (84.0%)	51.361	<0.001
	Yes	128 (33.1%)	97 (50.3%)	31 (16.0%)		
Week 2	No	266 (68.2%)	102 (52.3%)	164 (84.1%)	45.451	<0.001
	Yes	124 (31.8%)	93 (47.7%)	31 (15.9%)		
Week 3	No	252 (69.4%)	89 (50.6%)	163 (87.2%)	57.206	<0.001
	Yes	111 (30.6%)	87 (49.4%)	24 (12.8%)		
Week 4	No	272 (70.8%)	126 (59.7%)	146 (84.4%)	28.020	<0.001
	Yes	112 (29.2%)	85 (40.3%)	(15.6%)		

Significant differences in commuting type were observed between two groups in the first and second weeks. The intervention group reported higher levels of public transport use (57.7% in intervention vs. 48.1% in control in week 1, and 63.1% in intervention vs. 51.6% in control in week 2).

For sport type, there were significant differences between the two groups each week. Compared to the control group, the intervention group reported higher level of walking, jogging and other sport types.

For sport venue, there were also significant differences between two groups across all 4 weeks. The intervention group reported lower levels of sport venue problems—which means they were able to more effectively access sport venues to participate in PA.

Except during week 3, participants from the intervention group reported more positive emotion compared to the control group (63.9% in intervention vs. 46.9% in control in week 1; 61.3% in intervention *vs*. 37.4% in control in week 2; 49.3% in intervention vs. 29.4% in control in week 4).

Fast breathing and sweating were used as a subjective measure of PA intensity. Compared to the control group, this metric was significant higher level in all 4 weeks in the intervention group.

**Figure 3:**
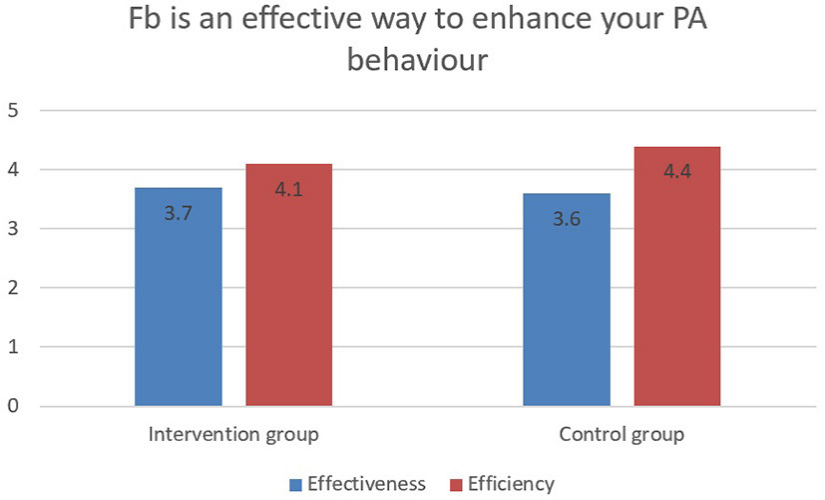
The effectiveness and efficiency of using Facebook to enhance PA behavior between two groups.

When participants were asked about the effectiveness and efficiency of using Facebook to enhance their PA behavior, the majority of the intervention group (77.4% or 24 out of 31) considered Facebook an effective way to enhance their PA behavior, compared to only 25.8% (8 out of 31) in the control group (Figure 3).

**Figure 4:**
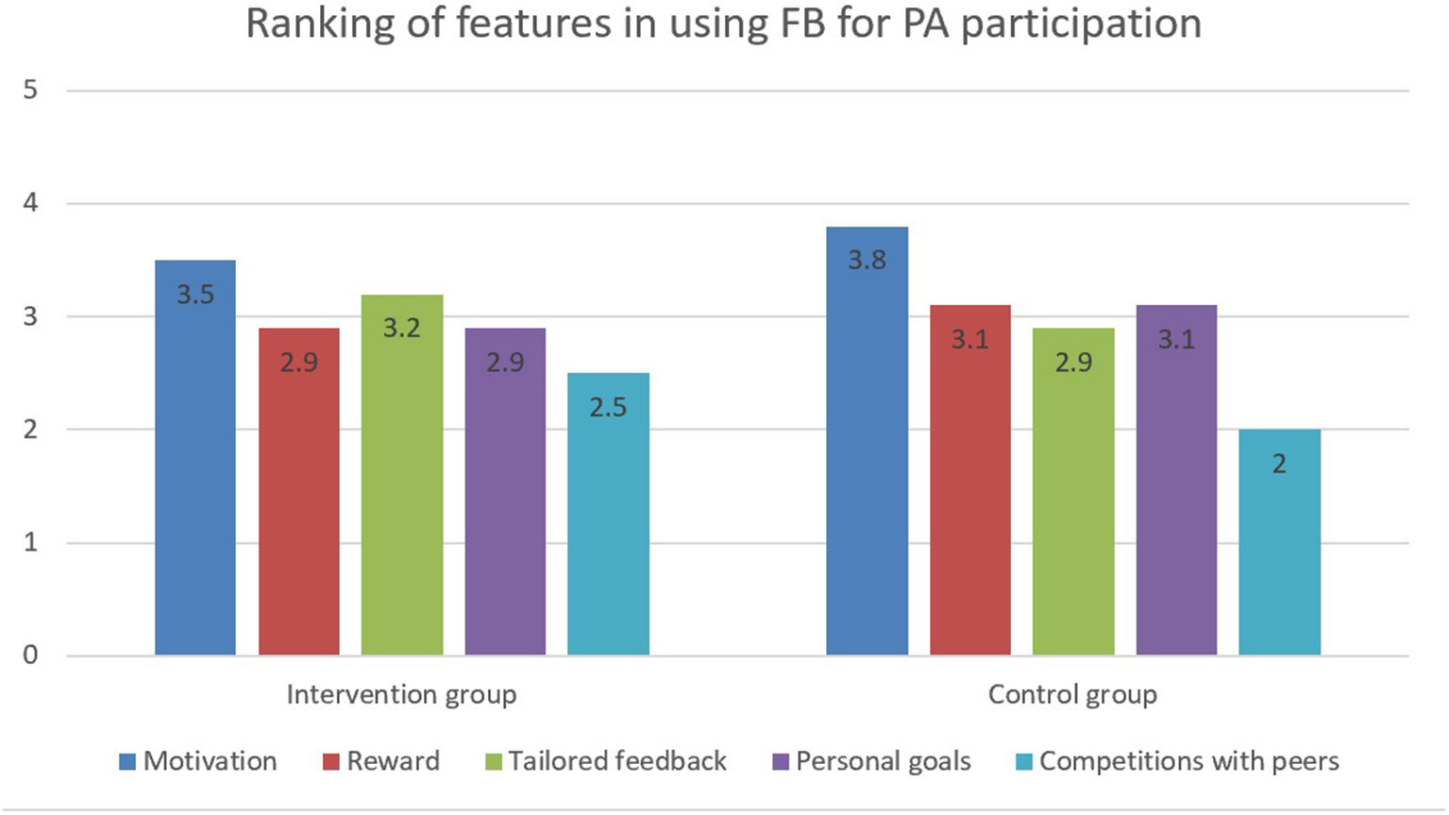
Ranking of features in using Facebook for PA participation between two groups.

Finally, the participants were asked to rank the most important features in Facebook that facilitated their PA participation. Motivation (supports from your friends) and tailored feedback (the responses from your friends are really personal and fits you) were the top two features listed by the intervention group, whereas motivation (supports from your friends), rewards (number of “Likes” you received) and personal goals [announcing your goal (s)/ progression to your friends] were the top three features in control group (Figure 4).

## Discussion

The most important findings from Study 1 were that behavior modification strategies (goal setting and self-monitoring), influence agents, snowball grouping and group size, social support (e.g., motivational quotes, interactive posts, and opinion polls), tailored feedback, and educational information are effective approaches for producing changes in PA outcomes in young adults *via* a Facebook intervention.

Based on the aforementioned recommendations, a Facebook PA intervention was designed, implemented, and evaluated using both objective and subjective measurements (Study 2). After a 4-week intervention, no significant differences were found between the intervention and control groups in PA level, as measured objectively with an ActiGraph. Other findings, including pedometer readings and more subjective assessments of PA were more favorable for the intervention group. This finding is consistent with previous studies that concluded that objective PA level did not increase significantly (Table 1) (25, 32, 42). However, this study did demonstrate a unique finding that perception of PA level (subjective) and positive emotions (happy and relaxed) associated with PA were higher (Table 6) during 3 of the 4 weeks in the intervention *vs*. the control group (Figure 2). This finding is meaningful because previous studies consistently indicated a deviation or discrepancy between actual and perceived PA level before and after an intervention. A classic review of attribution and perception of behavior change was conducted by Kopel and Arkowitz (43), who found that an individual's perception (including overt behavior, situational circumstances and physiological states) may have a significant impact on his/ her future behavior change and maintenance. This finding is linked to the “Think-Feel-Do” approach in cognitive behavioral therapy (CBT), which has produced numerous significant and effective treatment outcomes when linked with an individual's PA behavioral change in obesity management (44, 45).

From this study, the finding of subjective and positive perception of behavioral change in PA level indicated that the participants in the intervention group did “Feel” they have changed and increased their PA level but the “Do” stage has not been achieved yet. In the CBT triangle, “Think-Feel-Do” is a reciprocal relationship which reinforces each other in the cycle. The perceived change “Feel” is a reflection of what the participants attributed, and this attribution could act as an important determinant to actualize their behavior “Do” (43). Dijksterhuis and Bargh (46) explained the relationship between perception and behavior. They stated that perception provided human beings with an understanding of their environment. This understanding acted as a mean to respond to the environment and then translate into behavior. This process is named to be “Perceiving leads to doing.” Dijksterhuis and Bargh (46) concluded that perception produced or is linked to a drive to action. The finding from this study indicate that the perception of positive PA change is a meaningful transitional stage that deserves further investigation to link “Feel” to “Do.”

With regards to the categorical variables included in this study, we found that compared to the control group, the intervention group participated in a higher level of public transport in the first 2 weeks. The intervention group also reported higher level of walking, jogging, and other sport types, and significantly higher level of breathing fast and sweating in all of the 4 weeks. No previous studies have investigated the relationships between an individual's commuting pattern, sport types, sport venue, barriers, and exercise intensity and the use of a Facebook social network group. As a whole, these four variables may represent an individual's PA lifestyles and choices. Further investigation of how Facebook influences these lifestyles and choices may shed light on how to facilitate more objective changes in PA behavior.

Compared to the control group, the effectiveness and efficiency of using Facebook to enhance participant's PA behavior was rated much higher in the intervention group (77.4% vs. 25.8%). The different rating of effectiveness and efficiency may originate from the daily intervention message (goal setting, peer support, motivation, and tailored feedback) delivered to the intervention group by the captain. Rayward et al. (28) assessed the reach and new member registration rates resulting from a dedicated 10,000 Steps social media campaign by using Facebook. Results demonstrated that Facebook was a promising tool in promoting awareness and recruiting individuals to the intervention program, and it functioned as a successful long-term engagement strategy. Van Woudenberg et al. (47) also suggested that social network interventions may increase perceived social norm toward PA, and the use of influence agents may be the key to enhancing the effectiveness and efficiency of any PA behavior change. The previous studies have suggested that Facebook intervention can significantly increase PA behavioral adoption and engagement (13, 25, 27, 28, 48). This study further confirms this function in the social network and PA intervention.

Both intervention and control groups considered motivation (supports from your friends) as the most important feature when using Facebook to promote PA participation. Our findings confirm that peer support plays an important role during young adult's PA intervention. Allen (49) and Green (50) investigated the social reasons for youth sport participation and found that wanting to be part of the team with peers, perception of belonging, and identity formation facilitate significant motivation for PA participation. Most youth live in a collective environment, where companionship has a great influence. This motivation support from peers deserves further investigation to understand the exact content and impact when compared with an adult's health-oriented rationale for PA participation. Clearly, youth may have a different focus or rationale for choosing PA compared to adults. The primary motivators for adults (e.g., health benefits) may be obligatory factors for younger individuals (6). Therefore, guiding youth in accordance with their aptitude is likely important. For example, a qualitative study to examine the feasibility of Facebook to increase PA in teenage girls found that lifestyle activities such as walking and housework, welcomed by adults, were unfamiliar and unappealing to them. According to a social motivation and youth sport participation study by Tammelin et al. (51), youth are inclined to participate in more intensive sports such as different ball games, orienteering, track and field, cycling, gymnastics, skiing, etc. These sports require more diversified and advanced sports skills, and ultimately will lead youth into an adult sports world. Green (50) suggested that youth may invest their sport habits to accumulate social and leisure capital in which sport tastes, skills and interests are built for their lifelong use in the adult world.

Although many important findings have been reported, some limitations are worth mentioning. First, the sample size is small, which may affect the statistical power to detect an intervention effect. Since reachability is one of the advantages of social media compared to traditional intervention delivery channels, additional studies with larger sample sizes are warranted. In addition, the duration of the study was of 4 weeks. It is possible that a longer intervention duration is necessary for more permanent behavior changes. A third limitation is that participants may have changed their behavior (or reported changing their behavior) because they were being observed (e.g., the “Hawthorne effect”). It is important to continue to test Facebook interventions and their impact on PA behavior with attention to minimizing the Hawthorne effect when conducting intervention research (e.g., selection bias, differences in attention given to participants, primary or secondary observer effect, etc.). Finally, we did not collect data related to level of social media engagement (e.g., how much time participants spent on Facebook, how many comments they left, details of their comments, etc.). These data are important so that more specific elements of effectiveness can be examined.

## Conclusion

Study 1 identified the important features of successful Facebook interventions to increase PA in the literature. Some of the components related to the successful interventions included behavior modification (e.g., goal setting and self-monitoring), influence agents, the snowball method of recruiting, social support (e.g., interactive posts, motivational quotes, opinion polls), tailored feedback, and educational information.

Study 2 designed, implemented, and evaluated a Facebook PA intervention for young adults. Study 2 found that objectively measured PA did not differ between the control and intervention group, but that subjective perception of PA level and positive emotion were reported more frequently by the intervention than the control group, as were differences in commuting type, sport type, sport venue, and exercise intensity. Not surprisingly, the effectiveness and efficiency of using Facebook to enhance participant's PA behavior was rated much higher in the intervention group, and strategies preferred were slightly different. Using influence agents shows promise for facilitating higher levels of program engagement.

Although this study is focused on Facebook, the findings have meaningful implications for other online social network or e-health interventions. This study helps us understand more about using social networking technology to facilitate PA.

## Data availability statement

The original contributions presented in the study are included in the article/supplementary material, further inquiries can be directed to the corresponding author.

## Ethics statement

The studies involving human participants were reviewed and approved by Research Ethics Committee of Hong Kong Baptist University. The patients/participants provided their written informed consent to participate in this study.

## Author contributions

PL and JW designed the study, analyzed the data, and wrote the manuscript. PL collected the data. LR and LS and all other authors reviewed and revised the manuscript. All authors contributed to the article and approved the submitted version.

## Funding

Research funding was provided by the Fundamental Research Funds for the China Institute of Sport Science (Grant No. 20–14).

## Conflict of interest

The authors declare that the research was conducted in the absence of any commercial or financial relationships that could be construed as a potential conflict of interest.

## Publisher's note

All claims expressed in this article are solely those of the authors and do not necessarily represent those of their affiliated organizations, or those of the publisher, the editors and the reviewers. Any product that may be evaluated in this article, or claim that may be made by its manufacturer, is not guaranteed or endorsed by the publisher.
